# Understanding the Potential of Genome Editing in Parkinson’s Disease

**DOI:** 10.3390/ijms22179241

**Published:** 2021-08-26

**Authors:** David Arango, Amaury Bittar, Natalia P. Esmeral, Camila Ocasión, Carolina Muñoz-Camargo, Juan C. Cruz, Luis H. Reyes, Natasha I. Bloch

**Affiliations:** 1 Department of Biomedical Engineering, Universidad de los Andes, Bogotá 111711, Colombia; d.arango@uniandes.edu.co (D.A.); af.bittar@uniandes.edu.co (A.B.); np.esmeral@uniandes.edu.co (N.P.E.); c.munoz2016@uniandes.edu.co (C.M.-C.); jc.cruz@uniandes.edu.co (J.C.C.); 2 Grupo de Diseño de Productos y Procesos, Department of Chemical and Food Engineering, Universidad de los Andes, Bogotá 111711, Colombia; c.ocasion10@uniandes.edu.co (C.O.); lh.reyes@uniandes.edu.co (L.H.R.)

**Keywords:** gene editing, CRISPR/Cas, Parkinson’s disease, delivery vehicles, nanostructured materials, clinical trials, gene therapy

## Abstract

CRISPR is a simple and cost-efficient gene-editing technique that has become increasingly popular over the last decades. Various CRISPR/Cas-based applications have been developed to introduce changes in the genome and alter gene expression in diverse systems and tissues. These novel gene-editing techniques are particularly promising for investigating and treating neurodegenerative diseases, including Parkinson’s disease, for which we currently lack efficient disease-modifying treatment options. Gene therapy could thus provide treatment alternatives, revolutionizing our ability to treat this disease. Here, we review our current knowledge on the genetic basis of Parkinson’s disease to highlight the main biological pathways that become disrupted in Parkinson’s disease and their potential as gene therapy targets. Next, we perform a comprehensive review of novel delivery vehicles available for gene-editing applications, critical for their successful application in both innovative research and potential therapies. Finally, we review the latest developments in CRISPR-based applications and gene therapies to understand and treat Parkinson’s disease. We carefully examine their advantages and shortcomings for diverse gene-editing applications in the brain, highlighting promising avenues for future research.

## 1. Introduction

The advent of simpler, accessible, and less expensive genome editing technologies such as CRISPR-Cas9 has opened up immense possibilities for research into the genetic underpinnings of complex neurodegenerative diseases, such as Parkinson’s disease (PD), and the development of genetic therapies to treat them. PD is a complex and heterogeneous neurological disorder characterized by Parkinsonian motor symptoms (muscle rigidity, hypokinesia, tremors, and impaired gait) that progress in time, leading to more severe movement disorders as the disease progresses [1]. Moreover, PD can alter cognition, sleep patterns, and autonomous function, generally before motor symptoms appear [2]. PD symptoms are typically caused by a specific cellular process disfunction, such as the early death of dopaminergic neurons in the brain’s *Substantia nigra* pars compacta (SNpC). In general, cellular dysfunction and the death of dopaminergic neurons result from α-synuclein (α-syn) aggregation into Lewy bodies. It is now well understood that PD pathological hallmarks result from a complex interplay between genetic and environmental factors (idiopathic origin). Only in a minority of PD cases (approximately 10%), PD can be associated with genetic factors and runs in families (familial PD) [1,3,4,5]. These familial PD cases have been attributed to mutations in genes such as *SNCA* (encoding for α-syn), *PRKN*, *PARK2*, *PINK1*, and *PARK7* genes.

Genes identified in studies of familial PD have become candidates of interest for potential therapeutic intervention [2,5,6]. Additionally, these genes have been the focus of studies to better understand the biological routes disrupted in cases of idiopathic PD, likely caused by a combination of complex multigenic components and environmental triggers [5,7]. Understanding the genes involved in developing familial and idiopathic forms of the disease could elucidate the functional routes altered in PD and potentially guide novel treatments.

Based on the cellular processes implicated in PD, multiple treatment options have been investigated. Treatment targets and strategies can be classified as non-disease-modifying or disease-modifying depending on whether they aim to ease the disease’s symptoms or treating its underlying causes. Available treatment options for PD include only non-disease-modifying ones, targeting the dopaminergic function to control symptoms, and slowing down the disease [8,9]. These therapies either increase dopamine concentration or stimulate the brain’s dopamine receptors [1]. Treatment options remain limited and can lead to serious adverse side effects, such as dyskinesias, insomnia, and hallucinations, and can lead to additional complications over long-term use [10]. These limitations have motivated significant efforts to develop disease-modifying therapies that could slow down or stop the underlying disease’s progression. Efforts have focused on understanding new pharmacological targets for disease-modifying treatments [2], including targets for gene therapy [6] and the use of novel techniques such as cell transplantation (e.g., stem cell therapies) [11] and surgical interventions required for the administration of treatments to the target brain’s cell populations [2].

Gene editing has recently emerged as an innovative disease-modifying treatment alternative based on editing gene sequences or altering their expression profiles [2,5,6]. Gene therapies have the potential to permanently fix the underlying causes of the disease, which is not yet possible for most neurodegenerative diseases [12,13,14]. For more than two decades, site-directed genome editing has been possible using various molecules capable of recognizing and cleaving specific DNA sequences, such as zinc-finger nucleases and transcription activator-like effector nucleases (TALENs) [15]. Although these techniques represented the beginning of precise genome editing and its incursion into medical treatments, they are costly and challenging due to the complex nuclease design process [16]. A major revolution in genome engineering came with discovering CRISPR/Cas systems and their direct application as gene-editing platforms in mammalian cells [17].

Gene therapies based on CRISPR/Cas9 have already reached the clinical trial stage for many monogenic diseases, including sickle cell disease, b-thalassemia, and Leber congenital amaurosis [18,19,20]. Several of these therapies are currently in advanced preclinical testing stages, including the ones for Duchenne muscular dystrophy, hemoglobinopathies, and hereditary tyrosinemia type 1 [21]. Numerous gene therapies have also been tested to treat neurodegenerative diseases, including Alzheimer’s, metachromatic leukodystrophy, and spinal muscular atrophy [22,23,24,25]. The unique pathophysiology involving genetic, epigenetic, and idiopathic causes for each condition must be carefully evaluated to determine whether gene therapies are appropriate and which are the best strategies to implement them [26].

Before their widespread application in humans, several challenges and limitations must be overcome to ensure future therapies’ safety. Further research is required to address off-target modifications and rapid degradation and to develop solutions for addressing adverse reactions such as DNA damage [27] and immune responses [28]. The development of proper delivery vehicles could be a way to address some of the current challenges associated with CRISPR gene-editing applications [29]. Delivery vehicles based on nanostructured materials (both organic and inorganic, as addressed later) have emerged as powerful agents to internalize cells effectively and thus increase the efficiency of gene-editing technologies [30].

Here, we start by reviewing knowledge accumulated to date about the genes associated with PD, focusing on the different pathways known to become disrupted with the idiopathic form of the disease. We also examine how CRISPR-based gene therapies could be used as treatment alternatives for PD. In line with this, we review CRISPR techniques, their limitations, and the different types of vehicles that can be used to deliver the gene-editing components efficiently and safely. Finally, we review the current state of gene therapy-based clinical trials to treat PD [6].

## 2. The Genetic Basis of PD

PD is considered one of the most impactful neurodegenerative diseases, with an incidence of 17 per 100,000 persons per year [31]. Recently, significant research efforts have been devoted to developing disease-modifying alternatives based on gene therapies [32] targeting the genes and mutations that have been associated with familial Parkinson’s disease (Table 1). Consequently, understanding the genetic basis and the cellular pathways that become altered in PD is critical to identify gene-editing targets and ultimately develop effective gene therapies.

### 2.1. Genes at the Basis of PD

Familial PD has a more evident genetic basis than its idiopathic counterpart, facilitating the identification of genes underlying the disease and the ubiquitous pathways that might become altered in PD. Multiple genes involved in significant biological functions have been identified and studied in affected families (Table 1). Genome-wide association studies (GWAS) have been carried out to identify genes involved in PD and, with them, the biological pathways that become disrupted [33]. With this information, it has been possible to propose treatments targeting the affected biological routes and treat each PD patient according to their specific gene variants [34,35]. Most gene variants associated with familial PD are summarized in Table 1 [36,37,38].

**Figure 1:**
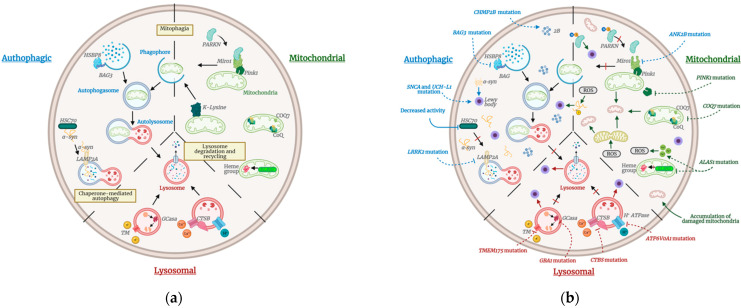
(**a**) Schematic of the normal autophagic, mitochondrial, and lysosomal pathways. The tight relationship between these pathways that become altered in PD enables the recycling of deficient organelles and degradation of outer material. Black solid arrows show the normal biological pathway. (**b**) Schematic of PD disrupted pathways. Dotted arrows indicate an up-regulation of the biological pathway specified due to variants in the genes involved. Meanwhile, inhibiting dotted lines indicate the down-regulation/inhibition of the biological pathway specified due to gene variants. Colored solid arrows show biological pathways that become disrupted in PD. Accumulation of damaged mitochondria and Lewy bodies due to dysfunctional lysosome degradation generates the typical PD physiopathology. Designed with biorender.com.

Idiopathic PD is characterized by late-onset age, slow progress, and prominent cognitive impairment or dementia, mainly in processing and evoking information [39,40]. Despite its higher incidence, idiopathic PD is less understood than familial PD due to its symptom variability and complex combination of multigenic and environmental causes [41]. To date, research in diverse areas has identified three main cellular pathways that become altered in idiopathic PD: autophagy, lysosomal, and mitochondrial. Restoring the activity of these pathways through gene editing is a promising avenue for future research in our path to ameliorate the symptoms and progression of both idiopathic and familial PD. It is thus important to identify key gene candidates within these pathways as potential therapeutic targets. Below, we discuss these pathways and the genes studied in the context of PD. Although each pathway has particular alterations, they share common attributes, as shown in Figure 1.

### 2.2. Autophagic Pathway

Together with the proteasomal systems, the autophagic pathway is involved in removing damaged proteins and organelles in highly metabolic nondividing cells [42]. Lysosomes mediate this metabolic pathway, relying on enzymes responsible for protein degradation. The general process consists of forming a phagophore around the damaged elements to create an envelope known as an autophagosome [43]. Subsequently, this structure fuses with a primary lysosome (known as an autolysosome), in which the degradation process occurs, as shown in Figure 1a. A large number of genes encoding for lysosomal enzymes (also related to familial PD) and involved in transport to the lysosome, mitophagy, or other functions related to autophagy, have been associated with the detrimental alteration of this pathway [44].

Among these proteins, HSC70 is essential in the hydrolyzation cycle from ATP to ADP. Specifically, this is dependent on its conformational changes for nucleotide and protein binding [45]. Postmortem studies have provided evidence of decreased chaperone-mediated autophagy activity in PD, which has been associated with significantly decreased levels of HSC70 in the SNpC and amygdala. These findings demonstrate that chaperone-mediated autophagy can be reduced in PD’s brains, contributing to Lewy bodies’ formation and the pathogenesis of neuronal degeneration [46,47,48].

Autophagy disruptions in neurodegenerative diseases significantly impact protein misfolding and abnormal aggregation, impeding neuronal survival (Figure 1b) [49]. Neuronal death and Lewy bodies, protein inclusions of misfolded or non-functional α-synuclein (α-syn) proteins [4], characteristic of PD can be attributed to mutations in the *SNCA* gene degradation systems [50,51]. The SNCA protein is involved in several cellular processes, including autophagy, endocytosis, exocytosis, neurotransmitter vesicle cycling, and has recently been related to DNA repair mechanisms [52]. Along this pathway, mutations in the genes *LRRK2* and *UCH-L1* have been correlated with Parkinson’s cases by permanently blocking chaperone-mediated autophagy [53,54]. The mutated *LRRK2* gene interacts with the chaperone-mediated autophagy (CMA) receptor, LAMP2A, which disrupts the receptor’s multimerization, resulting in substrates accumulation, including α-syn. Therefore, *LRRK2* mutations are linked with defective autophagy and subsequent α-syn accumulation [44]. On the other hand, the *UCH-L1* gene encodes a protein of the same name, present in Lewy bodies. UCH-L1 is an integral part of ubiquitin-dependent proteolysis because it allows the recycling of polymer chains from ubiquitin to monomeric ubiquitin. Studies in mesencephalic rat cultures found that inhibiting this gene causes dose-dependent degeneration of dopaminergic neurons and the formation of positive cytoplasmic inclusion of α-syn [55].

GWAS performed on familial PD have contributed significantly to identifying genes involved in the autophagic pathway damage, where 17 novel risk loci were reported, including *CHMP2B*, *BAG3*, *ANK2*, and *KAT8* [56,57]. In the first place, *CHMP2B* is a gene encoding the charged multivesicular protein 2B with unclear function but thought to be related to the endosomal secretory complex required for autophagy [58,59,60]. Moreover, studies in murine PD models have demonstrated that excess protein 2B expression generates autophagy impairment, protein accumulation, and subsequent cellular death [61]. The *BAG3* gene encodes for the BAG protein involved in chaperone-assisted selective macroautophagy. BAG is of particular interest because it binds to the protein HSPB8, facilitating the elimination of proteins prone to mutations, whose accumulation is expected in PD [62,63,64,65]. An in vivo study performed in an *SNCA*^A53T^ transgenic murine model (expressing human A53T α-syn variant) and *MG132*-treated *PC12* cells that overexpress wild-type α-syn confirmed that *BAG3* plays a vital role in regulating the elimination of α-syn through macroautophagy [63].

Similarly, *ANK2* is a gene encoding for a polypeptide called Ankyrin-B, which binds membrane proteins to the actin/myosin system in the cytoskeleton [66]. Studies have shown that the *ANK2* gene’s phosphorylation events are involved in Parkinsonian neurodegeneration [67]. This gene interacts with PINK1/PARKIN target proteins such as MIRO1 or ATP1A2 and ANK2-derived peptides, which are known to be important autophagy inhibitors [68]. Therefore, phosphorylation of *ANK2* inhibits autophagy of organelles, including mitochondria (i.e. mitophagy). Finally, *KAT8* is a gene encoding for K(lysine) acetyltransferase 8, a protein involved in autophagic regulatory feedback loops because it is highly correlated to H4K16 acetylation [69]. Research has shown that the reduction in *KAT8* acetylation is associated with the low regulation of autophagy genes resulting in cell death on mouse embryonic fibroblast [70]. K(lysine) also regulates the PINK1-mitophagy [71], as shown by research conducted to identify autophagic flow modulators on a high-content image-based ARNip. Moreover, this gene’s relationship with PD is verified by experiments where inhibiting *KAT8* generates a decrease in the autophagic flow [72,73].

### 2.3. Mitochondrial Pathway

Mitochondria are essential for cellular function due to ATP production, calcium homeostasis, and apoptotic signaling, and mitochondrial damage is associated with many diseases such as Huntington’s, Alzheimer’s, and myalgic encephalomyelitis [74,75,76]. PD has also been associated with the accumulation of damaged mitochondria in the neuronal cytoplasm [77]. Due to their function, mitochondria are more vulnerable to stress and consequent dysfunction [78], thus need to be constantly renewed especially in cells that have lost their mitotic capacity. Mitochondrial autophagy disruptions, characteristic of PD, begin with a protein known as PINK1, which should be imported through the membrane into healthy mitochondria [77]. Deficient and depolarized mitochondria fail to carry out this process, thus accumulating PINK1 in their membranes, as shown in Figure 1b. Normally, PINK1 accumulation recruits PARKIN, a ubiquitin ligase that facilitates mitochondrial degradation by autophagic and proteasome mechanisms [77]. Mutations in these genes cause the failure of this process, leading to the accumulation of damaged mitochondria. Other mitochondrial proteins and pathways typically dysregulated in patients with PD include the molecular chaperone prohibitin, the OMM VDAC1 protein, the mitochondrial import protein Tom40, the serine protease HtrA2, and the mitochondrial complex I function in the SNpC [79,80].

Loss of mitochondrial integrity and functionality condemn the cell to apoptosis. Under oxidative stress conditions, increased mitochondrial fission (separation of one mitochondrion into two) contributes to mitochondrial damage and cellular energy metabolism failure (Figure 1b). Thus, oxidative stress contributes to well-documented PD dopaminergic neurodegeneration [81,82,83,84]. Environments rich in reactive oxygen species (ROS) trigger cell organelle dysfunction and post-translational modifications of α-syn, such as the phosphorylation of serine 129, nitration, and ubiquitination. These processes facilitate the formation of toxic oligomers [85]. Therefore, the release of cytochrome C into the cytoplasm induces an apoptotic pathway mediated by caspases. These enzymes are also involved in cell proliferation, cellular remodeling, cell fate determination, and immune responses [86]. Moreover, studies in PD patients and animal PD models revealed a positive correlation between neuronal loss and activated caspase-1, -3, -8, and -9 in SNpC [87,88,89].

Numerous studies have reported that the *PINK1/PRKN* pathway becomes disrupted in PD. This may also be induced in vitro after exogenously adding α-syn or chemical perturbation [90,91,92]. Specifically, in a PD patient’s brain, PARKIN is S-nitrosylated and sequestered into Lewy bodies, which leads to lower availability of soluble, functional PARKIN [92,93]. In contrast, the accumulation of PINK1 is blocked by the inactivation of *PRKN*. This is supported by the high levels of PARKIN substrates found in a patient’s midbrain tissue [94].

Recently, novel genomic regions associated with an increased risk of developing PD have been identified, including mitochondrial pathway genes such as *MCCC1*, *ALAS1*, *ANK2*, *COQ7*, *CTSB*, *GALT*, and *ATP6V0A1* [95,96,97]. GWAS analyses worldwide have reported the presence of these risk loci in the Chinese [98,99], Taiwanese [100], European and American populations [101], thereby supporting the role of the genes mentioned above on PD susceptibility in multiple genetic backgrounds. For example, studies have identified a genetic association between *MCCC1* (which encodes for methylcrotonoyl-CoA carboxylase 1 protein) genotypes and age at onset, motor progression, and the overall risk of developing PD [102]. *ALAS1* (aminolevulinic acid synthase) encodes a protein with the same name whose function is related to biocatalysis of the aminolevulinic acid [103], a non-proteinogenic amino acid that plays an essential role in neuronal survival. Variation in its concentration can cause morphological and functional changes [104], which have been thought to be associated with PD.

Mitochondrial disruptions affecting the internal structure of mitochondria have been associated with PD symptomatology [105]. *COQ7* is critical to maintaining mitochondrial integrity and CoQ synthesis [106]. CoQ is essential in the mitochondrial electron transport chain, and mutations in *COQ7* have been linked to PD development [107]. Ebadi et al. also demonstrated that the action of neurotoxin 1-methyl-4-phenyl-1,2,3 6-tetrahydropyridine (MTPT) could induce Parkinson-like symptoms, which acts by inhibiting complex I [108]. GWAS findings in the Chinese population further support this locus’s association with PD susceptibility [106,109].

### 2.4. Lysosomal Pathway

Lysosomes allow the breakup of old and unnecessary structures, relying on digestive enzymes [110]. The lysosomal pathway and its role in the degradation of protein aggregates have emerged as a critical PD pathway because of its close relationship with the autophagic pathway. Lysosomes are essential for α-syn degradation, which forms the deposits responsible for the Lewy bodies and Lewy neurites observed in PD [111]. Traffic disruption occurs when lysosomal genes are altered, leading to intra-lysosomal buildup followed by persistence of α-syn in neurons, which ultimately results in apoptosis, as shown in Figure 1b [96]. For this reason, the lysosome pathway and its associated genes have been related to PD, including variants of *GBA1*, *TMEM175*, *CTSB*, and *ATP6V0A1* genes [56].

The *GBA1* gene encodes for lysosomal hydrolase β-glucocerebrosidase (GCasa), responsible for glucose hydrolysis. This ceramide glucocerebroside protein has shown a strong association with an increased risk of idiopathic PD with aging [112] and early-onset Parkinson’s with a higher incidence of neuropsychiatric symptoms [113]. Studies have shown that populations with both homozygote and heterozygote mutations in the *GBA1* gene with no PD symptoms show deteriorated motor abilities, cognition, and olfaction [114]. Thus far, it has not been possible to define the exact mechanism by which *GBA1* mutations mediate PD’s pathogenesis. However, it is known that not all carriers of the identified gene variants develop PD, providing further evidence for the complex multigenic nature of PD [115].

The *TMEM175* gene, another PD risk locus [109], encodes for a transmembrane (TM) endolysosomal potassium channel. The *TMEM175* p.M393T variant alters lysosomal and mitochondrial function, increasing the aggregation of α-syn, as well as the potassium conductance and luminal pH stability. Recent studies have shown a relationship between GCasa reduced activity and alterations in the *TMEM175* gene due to loss of channel integrity. Nevertheless, other genes could also be associated with such reduction in activity since *TMEM175* p.M393T and *LRRK2* p.G2019S variations explain only 23% of the variance in the GCasa activity [109]. Variations of the *TMEM175* gene can also cause a reduction in glucocerebrosidase activity, a deterioration in the autophagosome’s clearance by the lysosome, and a significant decrease in the mitochondrial respiration processes, which have also been strongly associated with PD [57,116].

The *CTSB* gene, belonging to the mitochondrial pathway, generates multiple protein products, including cathepsin B (CTSB). Ming Man et al. show that the CTSB protein ignites the MCOLN1/TRPML1 calcium channel in lysosomes, which maintains the suppression of the TFEB transcription factor and decreases protein expression related to lysosomes and autophagy [117]. Additionally, single-nucleus sequencing on postmortem brains shows that the *CTSB* gene is only expressed in neurons and microglia, suggesting that the variants observed in *CTSB* might have a significant impact on these cell types [97,117,118]. Together, these findings indicate that PD most likely involves a significant reduction in the lysosomal protease function [97].

Finally, the *ATP6V0A1* gene is expressed in microglia and its cell precursors. It encodes for the α1 subunit of the H + vacuolar translocating ATPase, a heteromultimeric complex responsible for acidifying the compartment of the secreting pathway and the secretion of granules [119]. This is V-proton ATPase functions pumping protons into the luminal environment of the endolysosomal system. The loss of function generates an increase in lysosomal pH and the inhibition of lysosomes and phagosomes fusion in the endolysosomal pathway [120,121].

## 3. Advances in CRISPR/Cas Systems and Delivery Strategies

CRISPR/Cas are RNA-guided endonuclease systems first discovered as an adaptive defense mechanism of procaryotes and archaea against viral infections [122]. Compared to other gene-editing techniques, CRISPR/Cas9 technology is simple, flexible, and less costly, which has led to its increasing popularity [123]. DNA edits induced by CRISPR/Cas9 systems have contributed significantly to our understanding of diseases by allowing us to evaluate the role of gene candidates, creating relevant cell lines and animal models, and finally, it can be a powerful tool for efficient and safe gene therapies in the near future [21]. CRISPR/Cas9 systems have been further adapted to allow for many genome manipulations beyond site-directed gene editing and are now used to modify the expression of a gene by modifying a catalytically dead nuclease (dCas9) to perform precise base edits [124,125], known as CRISPR activation (CRISPRa) and CRISPR interference (CRISPRi) technologies [126,127]. Moreover, they have been further developed into tools for epigenetic research [128], gene location detection [129], or even modified RNA targeting [130].

### 3.1. CRISPR/Cas Component Design for Precise Gene Editing

The success and precision of CRISPR/Cas9 applications depend largely on proper sgRNA design for the intended gene target. Unspecified genome editing, also known as off-target editing, is a significant obstacle to overcome for an eventual translation of CRISPR technologies into clinical practice. Off-target editing occurs when the endonuclease binds and cleaves a genome region other than the expected one, thereby generating unintended mutations [131]. Off-target editing could occur by several mechanisms, including the unspecific coupling of sgRNA to unplanned regions of the genome, 5-base mismatch in the endonuclease-specific protospacer-adjacent motif (PAM)-distal sequence of sgRNA, and DNA methylation that impedes biding of Cas9 [132].

Multiple tools have been developed to design sgRNA and predict possible off-target effects in silico based on mismatches and bulge size [133,134,135]. RNA guides are designed according to the specific target organism, DNA-target sequence, PAM sequence, off-target effects, and the transcriptional start site (TSS) [136]. These tools use various algorithms to design sgRNA while examining the genome of the target organism to avoid the off-target caused by sequence similarity [137]. However, multiple sgRNA should be tested simultaneously for in vivo and in vitro studies due to possible unexpected outcomes [138]. Currently, further cutting-edge methodologies based on deep learning are under development to improve sgRNA design [139].

Off-target modifications remain possible even after careful sgRNA design. Zhang et al. [140] found that a sgRNA designed in silico with no predicted off-target activity still caused three silent mismatches in *Arabidopsis*, one of them in the proximal PAM sequence (8th nucleotide) and causing high off-target activity. Despite being located at a significant distance from the PAM sequence, the other two mismatches also had attributable off-target effects. Similarly, Kleinstiver et al. performed a study comparing wild-type SpCas9 and a SpCas9-HF1 variant and found that even one pair base mismatch in the proximal section of the sgRNA significantly diminished SpCas9-HF1 gene-editing efficiency, in comparison to the wild-type SpCas9 [141]. These findings are only a few examples highlighting the importance of optimizing the design of sgRNA and improving computational and experimental methods for off-target effects detection and quantification. This is important to mitigate the possible off-target effects of CRISPR-based editing technology.

In addition to sgRNA design, another critical feature for accurate and efficient genome editing is the intracellular bioavailability of the CRISPR elements. This refers to the proportion of gene-editing components able to bind to intracellular targets [142]. Rapid degradation of CRISPR elements due to changes in factors such as pH and electrochemical gradients impedes accurate genome editing. Changes in pH conditions may cause protein denaturation, affecting active sites of interest and inducing degradation reactions [143]. Similarly, reducing conditions, often found in diseased cells, may cleave dimer cysteine disulfide bonds that maintain tertiary and quaternary structures together [144]. Therefore, delivery carriers for CRISPR elements protecting them from rapid degradation can be an efficient solution for boosting the bioavailability in target cells [142,145,146,147].

### 3.2. Delivery Strategies for CRISPR Applications

**Figure 2:**
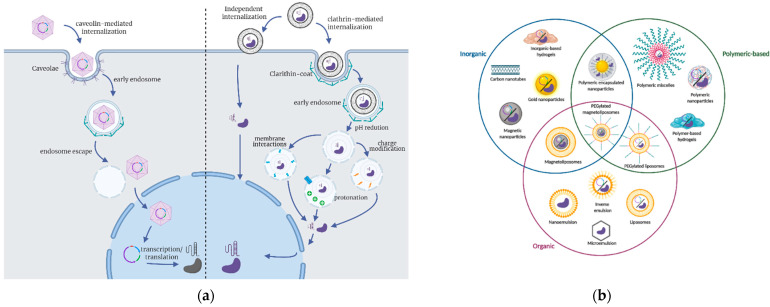
(**a**) Schematic of cell internalization pathways. Internalization can occur by three main mechanisms: clathrin-mediated, caveolin-mediated, and clathrin-caveolin independent internalization. The schematic shows two alternatives of CRISPR delivery: one using a viral vector for plasmid delivery (left) and liposomes for delivering RNP (right). The processes of maturation and endosome escape are also illustrated. Specific routes for endosome escape can vary for each delivery vehicle. (**b**) Schematic showing the nanostructured carriers currently used for delivery in CRISPR applications. Overall, nanostructured carriers can be divided into three subgroups: inorganic, organic, and polymeric-based vehicles. Similarly, nanohybrids are shown at the intersection of these families of nanostructured carriers. Nanohybrids of inorganic-inorganic materials were not included in this schematic. Alternative surface modifications can be incorporated to enhance the properties of each nanostructured vehicle shown, incorporating translocating proteins and pH-sensitive moieties. Depending on their unique characteristics, each delivery vehicle can also deliver either RNP or plasmid encoding for the CRISPR elements. Designed with biorender.com.

CRISPR component delivery strategies for gene editing can be grouped into three general approaches. First, the sgRNA and nuclease mRNA directly delivered into the cell/tissue/embryo. Second, sgRNA is delivered along with the synthesized nuclease protein called a ribonucleoprotein complex (RNP) [148]. Finally, the third strategy focuses on combining both sequences of sgRNA and nuclease in a single plasmid, which the target cell translates, as shown in Figure 2a [149]. The choice of delivery strategy entirely depends upon the characteristics of the specific CRISPR application. The delivery strategy for CRISPR elements can significantly impact the gene-editing success and the duration of the nuclease activity [150]. Each delivery strategy has different limitations and advantages that should be considered when choosing the CRISPR system of interest for the intended application [151].

RNP delivery has shown a lower off-target activity and faster genome editing than plasmid-nuclease delivery [152]. When choosing to deliver RNP directly into the cell, the specificity of CRISPR/Cas gene editing is highly dependent on nuclease purity, as well as its source organism. The preferred nucleases are from *Staphylococcus aureus* and *Streptococcus pyogenes* [131,153]. Recombinant nuclease alternatives have emerged in recent years to enhance editing specificity. Notably, modifications of Cas9 nucleases alter the nuclease-specific PAM sequences and ultimately enhance specificity while maintaining suitable editing efficiency. Studies have compared the activity of different Cas9 variants to find one that is optimal for gene-editing applications [154,155]. Some Cas9 variants such as wild-type SpCas9 and Sniper-Cas9 have high activity but low specificity; meanwhile, some other variants such as evoCas9 show higher specificity and therefore are optimal for avoiding significant off-target effects, even if there are slight mismatches between the sgRNA and the target sequence [155,156].

In contrast to RNP, plasmid-nuclease can generate a constitutive expression that leads to a more extended genome edition [157] and is a more stable and cost-effective alternative to RNP delivery. However, plasmid-endonuclease delivery is significantly limited by plasmid size, where a plasmid over 7 kb can pose significant delivery challenges [149,158,159]. This is especially relevant for some CRISPR applications where a tissue-specific plasmid design is required to address possible variations in the promoters [160]. Since designing a single plasmid vector with all the CRISPR components can be challenging, promising results have been obtained using separate plasmids with the nuclease and sgRNA sequences [161,162]. Finally, even though the delivery of RNA sequences encoding for nuclease is an attractive strategy, it can be difficult in some scenarios due to their rapid degradation [163].

The selection of delivery carriers for different applications can also have an enormous impact on the success of CRISPR gene editing because they directly influence the bioavailability of CRISPR components and gene-editing specificity. Recently developed nanoscale carriers have been shown to significantly improve plasmid delivery efficiency by altering the possible intracellular trafficking routes for the vehicle [164]. For example, PEGylated nanoparticles can increase efficiency by 40% compared to free plasmids in HeLa and NIH-3T3 cells [164]. The general mechanism of trafficking for a delivery carrier transporting CRISPR components follows four major steps: (i) cell internalization via numerous pathways including clathrin-mediated, caveolin-mediated, or clathrin-caveolin independent; (ii) maturation of the early endosomes; (iii) endosome escape by protonation, charge modification or specific membrane interactions (e.g., changes in endosome osmotic pressure, electrostatic adsorption on delivery carriers and insertion of dendrimers on endosome membrane); and finally, (iv) transfection of CRISPR components for later transcription/translation, as shown in Figure 2a [149,165,166].

### 3.3. Delivery Carriers to Improve the Performance of CRISPR/Cas9-Based Therapies

Even if all the components necessary to perform CRISPR gene editing can be delivered directly into a cell or tissue, using vehicles to transport RNAs, plasmids, and/or nucleases is advantageous to increase editing efficiency [29,167] and to reduce possible immune responses that can occur in the host due to random off-target integration into its genome [168]. Viral vectors are often used as delivery carriers. Unfortunately, they have multiple disadvantages, including activating a cyclic GMP-AMP synthetase, which could ultimately trigger apoptosis in some cell lineages [168,169,170]. Consequently, independently of the exact CRISPR chosen, a carrier is highly recommended to bring stability, prevent degradation, reduce off-target effects, and increase the target cells’ transfection levels [171]. Moreover, delivery carriers can avoid sgRNA and nuclease degradation even after passing through organ and physiological barriers such as the BBB, the extracellular matrix, and the P-glycoprotein transport system [158,172]. Some delivery carriers might escape endosomes efficiently and, consequently, lead to higher transfection levels and ultimately achieve genome editing efficiencies of up to 70% in different human cells [173,174]. CRISPR delivery carriers include viral vectors, lipid micelles, and nanostructured materials, as shown in Figure 2b [29,171]. Furthermore, there are several delivery strategies where the genetic material is directly incorporated into the target tissues by physical means [175,176].

Viral vectors were the first vehicles to achieve the successful delivery of CRISPR genome editing components. The most successful viral vectors are lentivirus, retroviruses, adenoviruses, and adeno-associated viruses [177]. In all cases, these carriers can deliver sequences of 4.5 up to 5 kb, which usually include the sgRNA and, depending on the virus, a small regulatory portion with information for promoter and polyadenylation sequences [172,178]. Multiple studies have repeatedly shown how viral vectors can be successfully used in gene editing [179,180,181], with numerous clinical applications [182]. Among viral vectors, adenoviral vectors’ have unique features, such as increased carrying capacity and non-pathogenic properties, that led to their increasing popularity in gene editing and CRISPR implementation [183,184]. AAVs are the preferred choice among adenoviral vectors, even if inflammatory reactions have been reported as a possible complication [185]. The use of AAV in multiple clinical trials demonstrated their safety and biocompatibility, leading to their approval by the FDA [180]. Although AAV insertion into the host genome is possible, most studies have shown that it is uncommon and targets the mitochondria. AAV is thus considered a safe approach [186], and it is frequently used as a delivery vehicle in gene-editing applications [187,188,189].

Some alternative AAV versions have been successfully tested to improve viral capacity and specificity. In general, modifications focus on altering the capsid proteins. In particular, AAV concatemers can drive long-term transgene expression due to their stable life cycle [161,180]. Peptides added in the VP3 region of the AAV genome generate vector re-targeting and thus enhance specific-organ transduction [190]. Consequently, modifications on the VP2 region can affect viral delivery efficiency and the transduction capacity of the vector [190]. Capsid engineering is a suitable route to accomplish specific requirements of a particular gene-editing application and can efficiently target the nervous system and crossing the BBB due to the tropism inherent to viruses [191]. For CNS gene editing, this modification is crucial, as transduction in CNS can fail due to the BBB’s limited permeability [192]. The BBB is adapted to filter molecules larger than 400 Da, and thus it can be a significant challenge for viral vectors to cross this barrier [193].

Viral vectors can also enable proper transduction processes for both dividing and nondividing cells in a wide variety of lineages, including hematopoietic, hepatic, and T-cells [145,172,194]. Viral vectors can accomplish simultaneous co-delivery of endonuclease and single or multiple sgRNA sequences for applications where multiplex knock-out might be needed, e.g., autism spectrum disorder, post-mitotic neurons, and schizophrenia [172,195,196]. However, viral vectors exhibit limitations in their loading capacity, preventing complex sequence delivery containing markers such as GFP and td-Tomato [162]. Likewise, long-term insertional activation mechanisms using CRISPR are difficult to control by viral vectors, thereby generating toxicity in some organs in vivo [194,197]. Moreover, AVV vector-based treatments can be relatively expensive and therefore are prohibitive for some CRISPR and gene delivery applications [197,198].

Lipid-based carriers for biotechnology applications are formed by a self-assembly process with amphiphilic molecules (with hydrophilic heads and hydrophobic tails) dispersed in aqueous solutions. In this process, the molecules’ hydrophilic heads come together to form a layer facing the aqueous solution while the hydrophobic tails stack together in a core [199]. These unique supramolecular structures or micellar systems are well-suited for encapsulation and transport cargoes with different physicochemical properties ranging from small hydrophobic drugs to large biological molecules, as shown in Figure 2b [200]. It is possible to create various delivery systems such as liposomes, lipid nanoparticles, and nano- and microemulsions [201]. Each system has its advantages regarding physicochemical properties that can be adjusted for different applications. For example, nano-emulsions are typically <300 nm and are kinetically stable, but microemulsions are thermodynamically stable and can present different geometries (e.g., worm-like, hexagonal, and liquid crystalline) [202]. To facilitate RNA and DNA loading, the carriers can be formed by incorporating cationic lipids as they readily form complexes with the negatively charged nucleotide sequences [199]. Therefore, lipid carriers are well-suited to deliver the plasmids containing the sgRNA and endonuclease sequences of CRISPR editing systems. Moreover, lipid-based vehicles have proven beneficial for delivering RNP or even co-deliver nucleic acids and proteins [29].

Lipid-based carriers have been successfully used in various applications, showing reduced off-target effects [203]. For instance, a self-assembled micelle delivery system for plasmid-nuclease delivery to induce human papillomavirus (*HPV*) E7 oncogene disruption led to a significant reduction in HVP cancerous activity. Cellular uptake was quantified using fluorescence-activated cell sorting (FACS), which showed a peak of Cas9 at 24 h. Thus, a fast turnover rate can be associated with low off-target effects in comparison to previous studies. CAS9/GFP expression increased by nearly 35%, eight hours after delivery using this F127/PPO-NMe3/pCAs9 micellar system in HeLa cells compared to controls, with no detectable off-target editing [203]. Other examples have proved the potential of nano-liposomal particles to deliver CRISPR systems [204,205].

The ease of synthesis and absence of immune response makes lipid-based carriers perfectly adapted to gene delivery applications [206], leading to their approval by the FDA in 1995 [207]. However, low colloidal stability and poor performance have limited their full implementation in CRISPR applications in the long term. Moreover, some studies have shown that lysosome degradation still occurs despite internalization, resulting in low gene-editing efficiencies [29]. Several chemical modifications can be used to overcome these limitations, including peptide and protein attachments, folate conjugation, and PEGylation [208,209,210]. PEGylation is the most popular alternative because it efficiently prevents enzymatic degradation, increases systemic circulation stability, and reduces clearance and charge-based contact with proteins and small molecules [211]. Another modification of interest is the conjugation of lipid-based carriers to metallic nanoparticles (e.g., gold and silver) to form complexes that favor circulation time due to controlled adhesion of plasma proteins and phagocytosis [212]. Recent in vivo studies with lipid-based carriers have demonstrated promising genome editing results. For instance, Cheng et al. developed selective organ targeting (SORT) nanoparticles based on charged lipids 18PA and DOPA for intravenous delivery of Cre recombinase mRNA in a td-Tomato murine model. Their results indicate accurate, independent genome editing in the spleen, lungs, and liver [213]. Lipid-based carriers have been successfully employed in multiple gene-editing applications [206,214].

Inorganic nanostructured carriers have emerged as potent delivery vehicles due to the consolidation of nanotechnology as a mature field to produce materials with highly controlled physicochemical properties, including size, morphology, crystalline structure, surface charge and chemistry, and 3D topology [29,215,216]. Moreover, their synthesis schemes have improved over time, resulting in high replicability, low costs, and simple synthesis routes. These carriers are also highly biocompatible and have chemical versatility, enabling surface conjugation with many molecules, including polymers such as polyethylene glycol (PEG) and polyacrylic acid (PAA) that increase stability, biocompatibility, and solubility by avoiding aggregation. Their potential application in the delivery of CRISPR technologies is not the exception since inorganic nanostructured carriers can carry high loads, improve the stability of the transported cargoes, and facilitate cellular uptake and specific sub-cellular targeting [146,217,218]. Additionally, it is possible to conjugate inorganic nanostructured carriers with different types of biomolecules superficially, such as antibodies, polymers, vitamins and proteins, and peptides [146,217,218,219] to improve cell penetration and endosomal escape. Functionalization of nanomaterials with translocating proteins or peptides such as OmpA, Buf-II, INF7, and GALA helps to escape an important fraction of endosomal compartments upon cell internalization, which can enhance transfection efficiencies in gene delivery applications [146,173,220].

Thus far, the most explored inorganic nanomaterials for drug delivery and gene-editing applications include carbon nanotubes (CT), gold nanoparticles (AuNP), silica nanoparticles (SiNP), magnetite nanoparticles (MNP), and graphene oxide (GO) [173,206,221,222]. More recently, nanohybrids have gained significant attention due to the possibility of enabling inorganic-inorganic, organic-inorganic, and bio-inorganic conjugation of materials, as shown in Figure 2b. Naturally, improved benefits for drug and gene delivery can be achieved due to the unique properties that each material brings to the nanohybrid [223]. Recently explored nanohybrids include magnetoliposomes, CdTe/ZnTe, and gadolinium-doped hydroxyapatite nanoparticles [224,225,226]. The possibility of manipulating the surface chemistry facilitates different conjugation strategies for the nucleotide sequences depending on responsiveness and colocalization monitoring as the molecules penetrate cells. Additionally, it is relatively straightforward to co-immobilize recombinant Cas9 at different ratios to optimize the editing process as needed and as a function of target cell line and expected off-targets [227]. Cell internalization and the subsequent intracellular localization of nanostructured carriers are primarily dependent on physicochemical properties such as size, surface charge, steric hindrance, morphology, and hydrophobicity [165].

Besides inorganic nanoparticles, polymeric nanoparticles have gained significant attention over the past few years due to their high stability, ease of synthesis and functionalization, high permeability through biological barriers, and solubility at physiological conditions [228,229]. Examples of polymeric nanomaterials for gene and drug delivery include polyethyleneimine (PEI), cyclodextrins (CD), poly-L-lysine (PLL), and poly(lactide-co-glycolide) (PLGA) [230,231,232,233]. In vivo studies have shown that these nanoparticles can be completely degraded in the body after 48 h in murine models [234,235], making them a sound delivery system for gene-editing applications.

Dendrimers have emerged as reliable drug delivery vehicles due to unique attributes such as monodispersity and well-defined chemical structure [236]. Moreover, due to their versatile building blocks (also known as generations) and superficial functional groups, it is possible to tune specific interactions (either electrostatic or covalent) with an ample variety of biomacromolecules (e.g., therapeutic proteins and polynucleotides). In the case of gene therapies where nucleotide sequences need to form a stable complex with the delivery vehicle, cationic dendrimers have been exploited to develop dendriplexes that can protect the genetic material from degradation. This approach is also favorable to overcome endosomal entrapment as this type of dendrimers can escape them by the proton sponge mechanism. This capability has been attributed to the amine groups in their structure with pKa values at or below physiological pH [237]. Some of the most valuable dendrimers that can be tailored according to the final application include polyamidoamine (PANAM), polypropyleneimine (PPI), poly-l-lysine (PLL), phosphorus (PPH), and linear-dendritic block copolymers (LDBC).

Various dendrimers have been designed and tested for brain targeting, and much effort has been invested in their surface engineering to assure that they can successfully come across the BBB while maintaining high biocompatibility, superior drug-release kinetics, and specificity toward the CNS cells [237,238]. For instance, hydroxyl-terminated PANAM dendrimers have demonstrated targeting of astrocytes and microglia as they pass through the BBB largely intact. Another issue of some dendrimers (e.g., cationic) is their high cytotoxicity, which exacerbates as the number of generations increases. This major hurdle has been addressed by conjugating highly biocompatible neutral or negatively charged moieties such as PEG, carbohydrates, and acetyl groups [239]. For gene-editing purposes in the brain, Taharabaru et al. tested PANAM dendrimers modified with cyclodextrins to deliver f Cas9 RNP both in SH-SY5Y cells and in vivo in eight-week-old BALB/c mice. Their results showed higher genome editing activity than Lipofectamine and Lipofectamine CRISPRMAX [240]. Dendrimer engineering with angiopep-2 peptide (which is capable of targeting LRP1 on the BBB) has also been exploited for higher delivery efficacy as it exhibits high transcytosis capacity and parenchymal accumulation [241]. This has also been the case of the RVG29 peptide, a 29-amino-acid peptide that stemmed from the rabies virus glycoprotein (RVG29), which targets the nicotinic acetylcholine receptor on the BBB [242]. The potency of dendrimers has also been exploited to develop multimodal therapies where small pharmacological molecules are co-delivered with gene therapies in search of possible synergistic effects. In addition, they have been coupled with polymeric nanoparticles (e.g., PLA and gelatin), quantum dots, and bacterial magnetic nanoparticles to form hybrid systems exhibiting unique structural and functional properties unattainable with the independent components [236].

An essential aspect of nanocarriers’ delivery is their surface charge, as it can directly alter their possible interactions with proteins and cell membranes. This, in turn, might lead to different routes for nanoparticle degradation in vitro and in vivo. In this regard, endothelial (HUVEC) cells and macrophages (Kupffer) have shown a marked tendency to internalize gold nanoparticles, which subsequently compartmentalize into endosomes and lysosomes [235]. Phagocytic degradation appears avoided when nanoparticles have a slightly negative surface charge [243,244]. In general, a low absolute surface charge, known as zeta potential, has been reported to prevent nanoparticle degradation both in vitro and in vivo. For instance, a recent study demonstrated that PEG-oligocholic acid-based micelle nanoparticles exhibiting high surface charge (zeta potential > 15 mV) showed a rapid degradation by macrophages and higher liver accumulation [243]. In contrast, nanoparticles with a slightly negative surface charge (<−8.5 mV) appeared to induce significantly less hemolytic and cytotoxic effects and reduced undesirable clearance by the reticuloendothelial system (RES) [243]. Finally, in SORT nanoparticles, slight changes in surface charge can lead to different organ targeting [213].

Another critical characteristic of nanostructured carriers is size and distribution. Recent delivery reports suggest that the proper size distribution of nanocarriers for drug delivery applications needs to be between 20 to 1000 nm [245,246]. Nanostructured carriers’ size is crucial to minimize clearance mechanisms, prevent aggregation, and avoid mechanical retention within capillaries. For instance, nanoparticles with average diameters <100 nm avoid sequestration by sinusoids in the spleen, while those with >20 nm evade kidney filtration [247]. Cell internalization of nanoparticles <200 nm is enabled by clathrin-mediated endocytosis, which needs to be cleared by nanocarriers to assure high gene-editing efficiencies [248]. Internalization through this mechanism starts by membrane events where clathrin-coated pills are recruited and eventually cleaved to form clathrin-coated vesicles. Then, these vesicles fuse with early endosomes and mature to lysosomes, where nanoparticles are either degraded by enzymatic action or are sent back to the cell membrane [249].

The last important nanoparticle characteristic that needs consideration is hydrophobicity, which has been linked to the route of nanomaterial degradation under physiological conditions where interactions with immunoglobulins and other plasma proteins are highly favored. As a result, changes in hydrophobicity make it possible to enhance capturing by the reticuloendothelial system (RES) [248]. Therefore, conjugating nanomaterials with hydrophilic polymers such as PEG, PLGA, poloxamer, chitosan, and poly (ethylene oxide) is a common strategy to avoid RES capture while improving biocompatibility [250]. Moreover, PEG polymers adsorbed on nanoparticles form a hydrophilic steric barrier that prevents clearance by macrophages or interactions with plasma proteins that ultimately triggers carriers’ clearance [197]. In vitro studies have also shown that some polymers might have a stabilizing role as demonstrated by conjugating Poly lactic-co-glycolic acid (PLGA), poly(oxyethylene), poloxamer, and chitosan [248], allowing PLGA nanoparticles to be highly stable under physiological conditions [251]. In addition to enhancing nanoparticle stability and bioavailability, hydrophilic polymers can also improve biocompatibility (most likely due to lack of recognition by the immune system) and cell uptake [252].

Nanocarriers can also be modified with stimuli-responsive moieties such that their cargoes can be delivered when nanocarriers reach specific organs or tissues [253]. For instance, our research group developed a pH-responsive nanocarrier combining a pH-responsive polymer (pDMAEMA) to core/shell magnetite/silver nanoparticles. These nanocarriers load and deliver plasmids in response to changes in the pH [220]. Other pH-sensitive nanocarriers that release their cargoes under reducing conditions further support the potential of these nanocarriers for the delivery of the CRISPR components [254].

Thus far, little is known about the use of nanostructured carriers for the delivery of CRISPR editing systems. Only a few studies have been conducted in vivo to estimate the true potential of delivery vehicles in enhancing transfection and on-target efficiencies. Among these studies, Lee et al. show that gold nanoparticles (AuNP) were able to deliver a CRISPR RNP system to successfully edit the *mGluR5* gene in the brain, rescuing exaggerated repetitive behaviors in a fragile X mouse model [29,95]. Wang et al. developed multi-AuNPs-lipid thermo-sensitive complexes to deliver CRISPR plasmids, achieving a release efficiency of 74% in mice [206]. Similarly, in vitro studies have used magnetic nanoparticles to introduce a complementary magnetically responsive adjuvant in a CRISPR application involving viral carriers, achieving a more precise genome editing [255]. This study proved that nanostructured vehicles could also be used for CRISPR editing systems as adjuvants to take advantage of their unique physicochemical properties. Although nanostructured vehicles have already enabled several gene delivery applications in recent years, issues regarding biosafety, wide particle size distribution, and low efficiency of surface chemical modifications are yet to be resolved prior to a full incursion pre-clinically and clinically [255,256].

Another important aspect to consider in CRISPR gene-editing applications is the delivery method for the vehicle carrying CRISPR components. Physical delivery is the most frequently used strategy for CRISPR systems in vitro and ex vivo delivery. This method dramatically increases the levels of readily available genetic material delivered [159]. Physical delivery standard techniques include microinjection, electroporation, and hydrodynamic delivery [29]. Although these techniques have not been specifically designed for in vivo studies due to cell structure damage, some reports have used physical delivery in zygotes to develop ex vivo transgenic animals [257]. Microinjection methodology was first used to generate transgenic animals by directly introducing DNA into the pronucleus [258]. On the other hand, electroporation is an alternative in which electrical currents alter the membrane potential. This creates transient nanopores to allow CRISPR elements to penetrate the cell by a concentration gradient [29]. Liang et al. achieved a 56% targeted integration efficiency for Cas9 RNP and ssDNA donors in HEK293 cells via electroporation, and precise genome editing rates of about 45% were reached in induced pluripotent stem cells (iPSCs) [259]. Compared with microinjection, electroporation has several advantages, including ease of implementation, its applicability in a broad range of cell types, simultaneous genome editing of different populations, and major genome editing in knock-out zygotes [260]. Lastly, hydrodynamic delivery takes advantage of the rise in permeability of specific cell types upon increasing the surrounding hydrodynamic pressure. This is performed by adding a large volume of solution to the target organs containing the CRISPR elements. Thus far, this approach has been the only physical delivery method used for in vivo testing of CRISPR systems [261].

Delivery carriers have recently emerged as excellent facilitators when using CRISPR systems for neurodegenerative disease research and treatment development. Encouraging results have been obtained using delivery carriers in Alzheimer’s, Huntington’s, and Parkinson’s disease research [262,263,264]. For instance, Park et al. developed nanocomplexes using the R7L10 amphiphilic peptide to deliver a silencing system of the *Bace1* gene, related to the accumulation of Aβ peptides, one of the main hallmarks of Alzheimer’s disease [262]. In Huntington’s disease, CRISPR/Cas9 has been used as a method for an early diagnosis using an amplification-free long-read sequence of the *HTT* gene [263]. Lastly, CRISPR application in Parkinson’s includes targeting the *LRRK2* gene, linked to familial PD. An in vitro study on hiPSC showed that an *LRRK2* knock-out led to a reduction in TH-positive neurons [264]. For detailed information on gene therapies to treat neurodegenerative diseases, an excellent review by Karimian et al. can be consulted [265].

### 3.4. Perspectives on Delivery Carriers and Potential for Future Research

CRISPR is a constantly evolving genome editing technique, and therefore, there is still much room to continue improving and developing new multifunctional vehicles capable of passing through different biological barriers to reach the intended target cells. This could also contribute to minimizing off-target effects such that the process of reaching clinical implementation might be accelerated. In addition to designing proper delivery vehicles, the main CRISPR components need to be carefully planned. As mentioned, an appropriate design of the sgRNA is critical for accurate genome editing, as is the choice of nuclease delivery strategy. Depending on the intended CRISPR applications, each of the nuclease delivery alternatives may be preferable for implementation by considering that, for instance, protein delivery leads to faster results than mRNA, but mRNA delivery provides a longer-lasting effect.

Delivery carriers enhance transfection and provide protection and stability for CRISPR components. Therefore, the latest CRISPR studies employ a wide range of delivery carriers primarily defined by the physicochemical properties that impact gene-editing efficiency. Nanostructured vehicles are the most promising carriers in gene delivery applications due to their chemical versatility. It is possible to modify their surface charge, chemistry, and hydrophobicity to increase stability and eventually avoid rapid lysosomal degradation. In the same way, nanostructures can be combined with other vehicles, adjuvants, or physical delivery strategies to maximize internalization and transfection while minimizing possible off-target effects synergistically. Unfortunately, in vivo studies with CRISPR systems delivered by nanostructured materials remain scarce [30]. Tight international legislation controlling the use of carriers in vivo and the contradictory results regarding their biocompatibility pose challenges that must be overcome before delivery vehicles can move into clinical applications. For now, the pharmacokinetics of delivery carriers remains poorly understood, and we need more research to further understand the various aspects involved in using nanocarriers in gene editing and therapies.

As expected, only a few CRISPR applications have been developed and successfully applied for PD treatment. Although CRISPR has been used to study the genetic causes of several diseases, it also offers a promising application as a gene therapy tool. Therefore, as a more comprehensive arsenal of delivery platforms becomes available, chances are high for developing safe and effective gene therapies that offer treatment alternatives to PD patients. For instance, a more comprehensive range of options for gene therapy will be readily available, along with the use of vehicles for applications that require gene knock-out, knock-in, repression, or activation.

## 4. Potential of Genetic Therapies as Treatment Alternatives for Parkinson’s Disease

Gene editing offers unprecedented potential to understand the molecular basis of PD, identify new treatment targets, and ultimately develop new gene therapies. Correcting dysfunctions in the biological pathways associated with PD can be accomplished by selectively editing or up- and down-regulating gene expression in key genes known to become altered in PD, including *GDNF*, *PINK1*, *PRKN*, or *AADC* [6]. Even if idiopathic PD has a complex multigenic basis and could not be treated by correcting variants in a single gene, gene editing could still represent a promising strategy to restore the activity of the key biological pathways that can become disrupted and cause PD symptoms.

**Figure 3:**
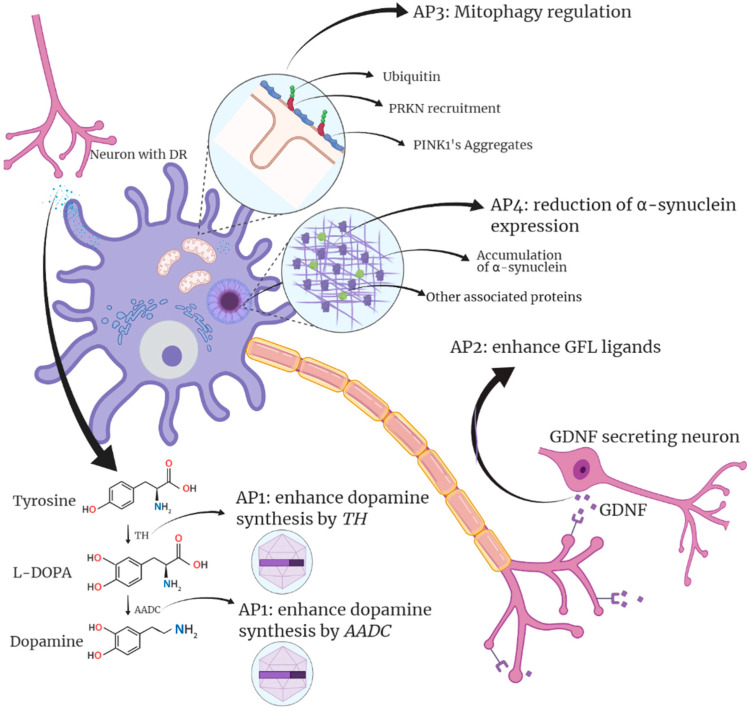
Summary of Parkinson’s gene therapy strategies and targets. The first approach (AP1) focuses on stimulating dopamine synthesis by targeting *AADC* and *TH* in dopaminergic neurons of the *Substantia nigra*. The second approach (AP2) focuses on GLF ligands. A third approach (AP3) focuses on the stimulation of mitophagy. Finally, the fourth approach (AP4) aims to reduce the presence of α-synuclein that ultimately generates Lewy bodies. Designed with biorender.com.

To date, gene-editing approaches for PD have been classified into four types according to the chosen therapeutic target [266]. An initial strategy aims to improve dopamine bioavailability in the brain. The second strategy focuses on neuronal regeneration by targeting neurotrophic factors. A third approach focuses on neuromodulation modifying genes in the subthalamic nucleus (STN). Finally, the fourth strategy is based on reducing α-syn production, thus ameliorating altered mitochondrial pathways [267,268,269]. All these approaches aim to modify the metabolic pathways involved in PD and neuron survival, mainly the aforementioned autophagic, mitochondrial, and lysosomal pathways (Figure 3).

The two pathways that have received the most attention in gene-editing studies related to PD are the dopamine pathway and neurotrophic factors. Manipulating neurotrophic factors can stop not only symptoms but also promote neuron survival. Alternatively, strategies involving the dopaminergic pathway mainly involve non-pulsatile stimulation of dopamine production that can drastically improve currently used treatments. The following sections will review gene editing and gene therapy advances in PD (summarized in Table 2).

### 4.1. Therapeutic Approaches Based on the Stimulation of Dopamine Production

Dopamine is a neurotransmitter involved in motivation, reward, pleasure, cognition, and motor control [285,286]. A dopamine deficiency detrimentally affects the neurons of the SNpC and is directly associated with PD symptoms (muscle stiffness, tremors, anxiety, and sleep disorders) [287]. Therefore, trying to recover this neurotransmitter’s normal levels is the aim of many currently available treatments. A typical strategy to normalize dopamine levels is via oral administration of levodopa (L-DOPA), which can cross the BBB, allowing an efficient treatment [288]. L-DOPA is a dopamine-precursor involved in the final step of the dopamine synthesis process. It is converted to dopamine through the decarboxylation performed by the aromatic L-amino acid decarboxylase (AADC) enzyme [275]. Even if these available treatments correct dopamine levels and may even offer some relief from PD symptoms, administered L-DOPA has low bioavailability (10%), and the fraction reaching the brain is only 1% [288]. In addition, using L-DOPA as a long-term oral treatment generate side effects such as dyskinesias and sleep disorders. [270,289,290]. Gene therapy offers an alternative route to increase dopamine production permanently by modifying the dopaminergic pathway and thus has received much attention in the last two decades.

Multiple studies have targeted the AADC (aromatic L-amino acid decarboxylase) [271] and TH (tyrosine hydroxylase) genes [270]. The TH gene encodes for the tyrosine hydroxylase responsible for converting tyrosine to L-DOPA during dopamine synthesis. Kirik et al. performed an in vivo study in a murine model where AAV was used to deliver the TH gene [270]. In this study, spontaneous and drug-induced behavior improvement was observed in rats with complete or partial 6-hydroxydopamine lesions of the nigrostriatal pathway, the bilateral dopaminergic pathway that connects the SNpC with the dorsal striatum [270]. The authors concluded that local intrastriatal TH delivery might be a viable therapeutic strategy in PD as it offers better control of orally administered L-DOPA’s adverse side effects. Further studies demonstrated the direct relationship between TH/GCH1 gene insertions and rats’ symptom improvement (behavioral recovery) [272]. The GCH1 gene, encoding the GTP cyclohydrolase 1 enzyme, is involved in the production of tetrahydrobiopterin (BPH4) and ultimately triggering dopamine production. However, the observed effect of editing the TH/GCH1 gene in murine models could not be replicated in other animal models [270,271], and the potential of modifying these pathways remains unclear.

Alternatively, it is possible to manipulate the *AADC* gene [291,292]. Gene therapy based on *AADC* modifications showed promising results in Phase I trials [275,293]. This therapy showed no complications in trial patients, was well tolerated, and no significant adverse effects related to the delivery vector (AAV) or the gene therapy treatment were observed [275]. Total and motor rating scales, quantified by Unified Parkinson’s Disease Rating Scale (*UPDRS*), improved over time for patients evaluated in the study. These results were corroborated by Muramatsu et al. [275,277].

Several Phase I studies have reported improvements in *UPDRS* for PD patients [276,291,292]. A comprehensive review on the use of *ADDC* in PD research was completed by Hitti et al. [266]. Despite all these promising results, Phase II trials are necessary to validate this approach’s safety and efficacy. Moreover, testing the use of delivery vehicles capable of crossing the BBB to avoid dangerous surgical procedures would be very useful, as these procedures can restrict clinical trials and in vivo studies [294]. Despite all these efforts, treatment alternatives focusing on dopamine stimulation remain a non-disease-modifying alternative [295].

### 4.2. Therapeutic Approaches Targeting Neurotrophic Genes

Current efforts to treat neuronal loss focus on glial cell line-derived neurotrophic factor (*GFL*) ligands, including glial cell line-derived neurotrophic factor (*GDNF*), neurturin (*NRTN*), artemin (*ARTN*), and persephin (*PSPN*). These neurotrophic factors are involved in the maintenance, survival, and differentiation of various neurons, including dopaminergic neurons [296]. As such, these genes represent important gene therapy targets to stop the progress of Parkinson’s disease and promote the generation of new neuronal tissue. Within this gene family, *GDNF* and *NRTN* have received the most attention. The therapeutic potential of *GDNF* for PD has been known for quite some time [297], as it has been associated with reduced neuronal development [298]. Attempts to develop therapeutic applications based on *GDNF* include the direct injection of the GNDF protein into the putamen, where results have shown a continued survival of lesioned nigral neurons for four months in rats [297] and monkeys [299]. These findings on animal models paved the way for Phase I clinical trials to evaluate the safety and efficacy of this type of therapy [300,301].

Contrary to expectations, Phase II trials yielded disappointing results. These studies concluded that gene editing on *GDNF* failed to confer a clinical benefit to PD patients [302]. It was uncertain whether technical differences between this trial and open-label studies contributed to this negative outcome [302]. Later research attributed this failure to inadequate diffusion of the GDNF proteins in the brain putamen [303]. New attempts to resolve this problem continue to emerge and have achieved protein diffusion throughout all the putamen [304]. Using nanostructured vehicles for delivery can be an alternative for boosting the bioavailability of proteins and improve clinical trial results [280].

Gene therapy presents a unique advantage to permanently correct PD abnormalities through modifications in the GDNF pathway, permanently increasing the production of GNDF. Chen et al. successfully evaluated this possibility showing that motor ability improved after gene editing in MitoPark mice, an animal model for PD [281]. In this novel study, the authors used macrophages that expressed *GDNF* for treatment and managed to increase the expression of GDNF up to three times compared to the control in plasma, SNpC, and striatum [278]. In addition, no evidence of side effects of macrophage-mediated GDNF therapy was detected in the study. This gene therapy evaluation was carried out parallel to the direct injection of the protein into the tissue, showing similar results to viral vectors [279,282]. Studies targeting GDNF for Parkinson’s disease treatment include various administration vehicles. For example, there are studies with encapsulated protein-producing cells [305], microspheres [283], cationic microbubbles [284], and BBB-penetrating nanoparticles (BPN) [306]. A more recent approach uses SINEUP-RNA delivered by AAV to increase the *GNDF* gene’s translation, demonstrating effective results in a PD mouse model by improving motor deficits and ceasing neurodegeneration [307].

The *NRTN* is another essential neurotrophic gene in PD, showing promising results as a therapy target. However, the therapy’s effectiveness was again disappointing in double-blind trials after initial validation of AAV-mediated *NRTN* delivery therapy in animals [308,309]. In Phase I trials [310], no differences were found between treatment and control groups [311]. Further analyses carried out postmortem 8–10 years after treatment revealed that *NRTN* had limited expression and coverage (~3%–12% of the putamen and ~9.8%–18.95% on the SNpC) in study patients. Moreover, it was determined that there was no difference in the degree of Lewy pathology between the treated group and untreated patients with Parkinson’s disease [312]. These findings are consistent with the results of previous clinical trials and currently halt further trials targeting the *NRTN* gene [310,311].

### 4.3. Other Gene Therapy Approaches

#### 4.3.1. Approaches Targeting Mitochondrial Genes

Among genes in mitochondrial pathways, *PRKN* and *PINK1* have been essential targets in PD research [313]. *PRKN* and *PINK1* have been mainly associated with genetic PD [314] but have been considered targets to improve mitochondrial pathway function in all PD types [315]. Currently, strategies focusing on mitochondrial pathways are scarce, and there are no Phase I trials evaluating strategies targeting these genes. However, progress has been made in understanding these genes’ role in the disease and the possible therapeutic implications for addressing their alterations [316]. One example is the study of Yan et al., who used gene-editing techniques to understand the mitophagy process in detail. They corroborated the direct relationship between damage in *PINK1* and *PRKN* with PD [317]. It has been shown that the natural replacement of damaged *PINK1/PRKN* genes generates protection against PD [267]. Koentjoro et al. demonstrated that *Nix* (Nip3-like protein X of the mitochondrial autophagy, a protein that induces autophagy) could function as a protective molecule, preventing a carrier of homozygous *PRKN* mutations from developing PD [267]. Using human fibroblasts, the study confirmed that *Nix* could facilitate mitochondrial clearance and, therefore, supports mitochondrial function despite the lack of the *PINK1/PRKN* pathway. Consequently, the *Nix* gene has therapeutic potential for PD treatment [267]. This approach can also provide a solution for idiopathic PD since proper regulation of these genes could favor a PD patient’s mitochondrial proliferation and health [315]. As shown by Chung et al., who developed PARKIN-permeable cells, its delivery increases mitophagy, mitochondrial biogenesis and suppresses α-syn accumulation in cells treated with mitochondrial toxins (sodium arsenite and rotenone) [317].

#### 4.3.2. Approaches Focused on α-Synuclein

Parkinson’s disease is characterized by the formation of Lewy bodies in neurons (see Section 2, [318]). Therefore, interference with the sequence and expression of the *SNCA* gene offers a promising alternative to control Parkinson’s disease progression [268,269]. Studies focusing on *SNCA* as a gene-editing target have demonstrated that its silencing in the hippocampus of mice using an AAV-delivered microRNA can significantly reduce the behavioral deficits associated with PD [268,269]. In a study performed by Ye-Han et al., AAV gene silencing vectors were designed to determine *SNCA* silencing’s efficiency and specificity against human α-syn (hSNCA) [319]. Although neurotoxicity was observed [319,320] in vitro, data suggest that miRNA-embedded silencing vector may be ideal for *SNCA* silencing. However, there is a long list of issues and challenges to address before thinking about these studies’ clinical translation. Finally, in a recent study, gold nanoparticle composites were loaded with plasmid DNA (pDNA) to inhibit α-syn expression [321]. The authors observed that nanoparticles improved TH levels and decreased aggregation of α-syn in SNpC. The nanocomposites attenuated motor dysfunction and reversed the inhibition of long-term potentiation (LTP). LTP is one of the main cellular mechanisms that appear to underlie learning and memory. These results indicated that the nanocomposites had significantly neuroprotective effects in motor and non-motor dysfunction in PD mice [321].

### 4.4. Stem Cell-Derived Therapies and Stem Cell In Vitro Models

Our current knowledge about the pathophysiology and biological pathways of PD has directed research strategies using human stem cells to replace and regenerate dopaminergic (DA) and other cells. There are three main strategies based on the source and type of stem cells: (1) fetal mesencephalic tissue, (2) mesenchymal stem cells (MSCs), and (3) pluripotent stem cells (hPSC), including embryonic stem cells (ESC) and induced pluripotent stem cells (iPSC) [322]. A thorough review of the use of stem cells in PD can be found by Liu and Cheung [322].

Recent years have seen increased interest in the use of gene editing in stem cells to better understand the molecular basis of PD. Stem cells derived from PD patients and induced pluripotent stem (iPSCs) are relevant in vitro models to study the molecular mechanisms of the disease and develop therapeutic strategies [323]. IPSCs are cells that resemble embryonic stem cells by delivering OCT4, Sox2, Klf4, and c-Myc factors via retrovirus vehicles to somatic cells [324]. Furthermore, iPSCs derived from PD patients all for the control of genomic background, providing a personalized model to directly establish [325] and differentiate [326] iPSCs into relevant DA cells and subsequently assess the impact of genetic mutations on disease development and severity.

Integration of the CRISPR/Cas9 systems and the iPSC model offers the possibility of manipulating pathogenic genes (turn off/on), and eliminating phenotypic differences caused by individual inheritance, thereby providing a more direct understanding of the relationship between specific genes and PD. This platform can provide a vast iPSC PD library that allows analyzing the effects of single nucleotide polymorphism (SNPs) and drug response differences [264]. These studies have allowed significant advances in our understanding of the relevant molecular events and mechanisms associated with this disease [327,328,329,330]. Human iPSC technology allowed a better approach to preclinical studies and provided promising clinical trials results [331].

Work on the use of stem cells to study and treat PD paved the way to the 2015 global task force (called G-Force-PD) aimed at bringing the hPSC and iPSC work to clinical trials and to share the obtained information publicly [332]. The possibilities offered by these cells that can differentiate into multiple neuronal lineages as well as their ability to develop into three-dimensional aggregates, known as organoids, combined with gene-editing strategies such as CRISPR, provide a suitable platform in which to study the complexity of the disease much more accurately. Moreover, the development and progression of the disease and the association with genetic mutations can be targeted robustly with these platforms [323]. Studies where these approaches have been explored and studied in detail can be consulted elsewhere [329,333,334,335,336,337]

### 4.5. Future of Gene Therapy for Parkinson’s Disease

Parkinson’s disease has a complex pathological framework, including multiple metabolic pathways associated with the degradation of substrates and the accumulation of harmful material in neurons’ cytoplasm. The characteristic complexity of idiopathic Parkinson’s and the underlying cause of neuronal loss explain why the initial triggers for the disease remain largely unknown. Strategies based on gene editing aimed to evaluate how the underlying metabolic pathways become disrupted will contribute to our understanding of PD at the molecular level. This knowledge will be vital in identifying new genetic targets to enable more robust and comprehensive treatments of PD.

To date, a great diversity of approaches to treat PD have been evaluated due to the disease complexity. Strategies aimed at increasing dopamine levels, improving neuronal survival, preventing damage in the mitochondria, and preventing α-syn aggregation. These approaches have been extensively studied in cellular and animal models, revealing their potential as targets for future disease-modifying treatments. Clinical trials, however, have revealed challenges and limitations to these new treatment avenues that are yet to be resolved. Significant limitations in new PD treatment development regard delivery issues, including inadequate diffusion of proteins, rapid degradation of delivery vehicles, and viral vector drawbacks. The design and testing of new carriers that enhance transfection and limit immune response, such as multifunctional nanostructured vehicles, have immense potential to overcome these challenges. Furthermore, CRISPR technology now offers an efficient, accurate, and potentially safe toolkit for gene editing (i.e., knock-in and knock-out) or transcriptional modifications (i.e., CRISPRa and CRISPRi) [338]. Delivery of CRISPR components with efficient and safe carriers offers a unique opportunity to correct the biological pathways that become disrupted in Parkinson’s disease, offering a new disease-modifying treatment alternative for both familial and idiopathic PD. More research is still necessary to identify the best gene therapy targets and develop better gene-editing vehicles for superior tissue selectivity, increased safety, and higher on-target edition rates.

## Figures and Tables

**Table 1:** Familial Parkinson’s genes identified until 2020. Details are presented on the proteins encoded by these genes, their function, the pathway they are involved in, and what type of Parkinson’s they are associated with.

Gene	Alternative Gene Names	Gene Locus	Protein	Protein Function and Cell Pathway Governed	Onset of Familial PD
*SNCA*	*PARK 1 or PARK4*	4q22.1	α-synuclein	Synaptic vesicles trafficking	Early
*Unknown*	*PARK3*	2p13	Unknown	Unknown	Late
*UCHL1*	*PARK5*	4p13	Ubiquitin C-terminal hydrolase L1	Proteasome system	Late
*LRRK2*	*PARK8*	12q12	Leucine-rich repeat kinase 2	Autophagy processing	Late
*HTRA2*	*PARK13*	2p13.1	HtrA serine peptidase 2	Mitophagy development	Unknown
*VPS35*	*PARK17*	16q12	Vacuolar protein sorting 35	Endosome regulation	Late
*EIF4G1*	*PARK18*	3q27.1	Eukaryotic translation initiation factor 4 gamma 1	Protein translation	Late
*DNAJC13*	*PARK21*	3q22.1	DnaJ heat shock protein family (Hsp40) member C13	Endosome regulation	Late
*CHCHD2*	*PARK22*	7p11.2	Coiled-coil-helix-coiled-coil-helix domain containing 2	Mitochondria-mediated apoptosis and metabolism	Late/Early
*PRKN*	*PARK2*	6q26	Parkin	Mitophagy development	Early
*PINK1*	*PARK6*	1p36.12	PTEN-induced putative kinase 1	Mitophagy development	Early
*DJ-1*	*PARK7*	1p36.23	DJ-1	Mitophagy development	Early
*ATP13A2*	*PARK9*	1p36.13	ATPase cation transporting 13A2	Lysosomal function	Early
*GIGYF2*	*PARK11*	2q36-7	GRB10 interacting GYF protein 2	Insulin-like growth factors (IGFs) signaling	Early
*PLA2G6*	*PARK14*	22q13.1	Phospholipase A2 group VI	Lipids metabolism	Early
*FBXO7*	*PARK15*	22q12.3	F-box protein 7	Mitophagy development	Early
*DNAJC6*	*PARK19*	1p31.3	DnaJ heat shock protein family (Hsp40) member C6	Endosome regulation	Early
*SYNJ1*	*PARK20*	21q22.11	Synaptojanin 1	Endosome regulation	Early
*VPS13C*	*PARK23*	15q22.2	Vacuolar protein sorting 13 homolog C	Mitophagy development	Early
Unknown	*PARK10*	1p32	Unknown	Unknown	Unknown
Unknown	*PARK12*	Xq21–q22	Unknown	Unknown	Unknown
Unknown	*PARK16*	1q32	Unknown	Unknown	Unknown
*GBA*	*-*	1q22	Glucosylceramidase beta	Lysosomal function	Late
*LRP10*	-	-	LDLR-related protein 10	Retinoid metabolism and transport	Late

**Table 2:** Summary of main research studies on neurotrophic factors and increased dopamine expression in the context of PD. DP: brain dopamine. AVV: adeno-associated virus, TH: tyrosine hydroxylase, AADC: aromatic L-amino acid decarboxylase.

Approach	Vector	Phase of Clinical Study	Reference
DP activity *(TH)*	AAV-TH	Animal Model: murine	[270,271,272,273]
DP activity *(AADC)*	AAV-AADC	Animal Model: murine (Phase I)	[274,275,276,277]
Neurotrophic genes *(GDNF)*	AAV-GDNF	Animal Model: murine and primate (Phase I and II)	[278,279]
Neurotrophic genes *(GNDF)*	Hematopoietic stem cell macrophages	Animal Model: murine	[280,281]
Neurotrophic genes *(GNDF)*	Encapsulated GDNF-secreting cells	Animal Model: murine	[282]
Neurotrophic genes *(GNDF)*	Cationic microbubbles	Animal Model: murine	[283]
Neurotrophic genes *(GNDF)*	Brain penetrating nanoparticles	Animal Model: murine	[284]

## Data Availability

The data underlying this article are available in the article.
